# Focusing Sensor Design for Open Electrical Impedance Tomography Based on Shape Conformal Transformation †

**DOI:** 10.3390/s19092060

**Published:** 2019-05-02

**Authors:** Yu Wang, Shangjie Ren, Feng Dong

**Affiliations:** Tianjin Key Laboratory of Process Measurement and Control, School of Electrical and Information Engineering, Tianjin University, Tianjin 300072, China; tju_wangyu@tju.edu.cn (Y.W.); fdong@tju.edu.cn (F.D.)

**Keywords:** open electrical impedance tomography, sensor design, conformal transformation, focusing sensor, open domain imaging

## Abstract

Electrical Impedance Tomography (EIT) is a non-invasive detection method to image the conductivity changes inside an observation region by using the electrical measurements at the boundary of this region. In some applications of EIT, the observation domain is infinite and is only accessible from one side, which leads to the so-called open EIT (OEIT) problem. Compared with conventional EIT problems, the observation region in OEIT can only be measured from limited projection directions, which makes high resolution imaging much more challenging. To improve the imaging quality of OEIT, a focusing sensor design strategy is proposed based on shape conformal theory. The conformal bijection is used to map a standard EIT sensor defined at a unit circle to a focusing OEIT sensor defined at an upper half plane. A series of numerical and experimental testes are conducted. Compared with the traditional sensor structure, the proposed focusing sensor has higher spatial resolution at the near-electrode region and is good at distinguishing multi-inclusions which are close to each other.

## 1. Introduction

Electrical Impedance Tomography (EIT) is a noninvasive imaging method, which reconstructs the conductivity distribution of the imaging field [1]. It applies an electrical excitation signal to the target field through electrodes placed at the boundary of an observation area, and then obtains the electrical responses reflecting the conductivity distribution within the observed field. This technology has the advantages of portability, low cost and high time resolution. EIT has been widely applied in the monitoring of many industrial and biomedical processes [2,3]. Conventional EIT aims at reconstructing the conductivity distribution within a closed domain [4,5,6]. However, in practical applications, the observed domain is not always closed or is big enough to be approximated to infinity compared with the size of the EIT sensors. This leads to the open EIT (OEIT) problem.

OEIT usually takes an open area with an unclosed boundary as observation field for measurements. OEIT was used in surface geophysics for surface geological exploration in an earlier time. Then it was also used in structural damage detection, landmine detection and biomedical tissue imaging. Youssef et al. [7] used OEIT to detect sinkholes in Saudi Arabia, and the results showed that the OEIT was effective in detecting and mapping a known sinkhole in the study area. Church et al. [8] constructed a prototype confirmatory landmine detector based on OEIT to image buried landmines. The results demonstrated that it is possible to reliably reconstruct conductivity perturbations of a shallow buried antitank mine or similar object in a variety of soils. Baltopoulos et al. [9] using OEIT to map damage in carbon fiber reinforced polymer plates. The methodology is validated based on experimental damage scenarios, successfully identifying the induced damage. Allen et al. [10] used an electrical impedance tomography-based sensing skin for quantitative imaging of damage in concrete. In the meantime, OEIT has been extensively studied in clinical medicine. Mueller et al. [11] developed an OEIT sensor with 4 × 4 electrodes for detecting conductivity changes during ventilation and perfusion. Time traces of the reconstructed conductivity distribution demonstrated the detected changes in conductivity were due to ventilation and perfusion. Borsic et al. [12] used a cylindrical probe based on EIT with an array of electrodes on the front surface to detect prostate disease. They also developed a novel reconstruction algorithm used for conductivity estimation. The simulation results demonstrated the feasibility of imaging moderately contrasting inclusions at distances of three times the probe radius from the probe surface. Cherepenin et al. [13] developed a 48-electrodes OEIT system combined with a vaginal probe for early detection of cervical neoplasia. They checked their system on a saline solution tank containing different small objects. The results showed the system could distinguish the size and location of single targets and the type for different targets. Aristovich et al. [14] used a 30-electrodes OEIT array to reconstruct images of fast neural-evoked activity in the rat cerebral cortex and developed a novel noise-based image post-processing technique. The results indicated that the developed methods may be expected to be reliably applied for imaging neural activity with planar arrays. Murphy et al. [15] used an end-fired microendoscopic EIT probe for surgical margin assessment and developed a novel regularization technique using the dual-mesh method. The results showed the feasibility of surgical margin detection using microendoscopic EIT.

OEIT thus has broad application prospects, however, reconstructing images in the open-domain geometry poses additional challenges compared with conventional EIT systems. In OEIT, the electrodes are usually arranged on one surface of the observation field, thus the current density decreases rapidly with the distance from the electrode surface. In the meantime, the incomplete boundary conditions also increase the difficulty of solving the OEIT problem, making the imaging quality of OEIT relatively low and sensitive to noise. Thus, there is still a large scope for the improvement for OEIT. Improved imaging algorithms can improve the reconstruction quality of OEIT to a certain extent, but algorithm improvement cannot fundamentally improve the spatial and temporal resolution of OEIT. This is because the electric field distribution in the observation field is fixed under the premise of not changing the electrode configuration and excitation strategy. The improvement of the imaging algorithm can only increase the acquisition of measurement information, but cannot improve the electric field distribution. However, different electrode configurations and data collection patterns will affect the penetration depth of the electric field, thus affecting the spatial resolution and imaging depth of OEIT. Perez et al. [16] designed a novel rectangular array of 20 active electrodes to inject the external currents, and 16 passive electrodes to measure the induced voltages. The results showed that this kind of electrode arrangement can avoid problems due to the unknown contact impedance. The reconstructions have good spatial resolution in the *xy*-plane and are quite stable with respect to the noise level in the data. Liu et al. [17] used a scanning linear electrode for the imaging of open domains and proposed a novel measurement and stimulation pattern. This research is dedicated to increasing the number of independent voltage measurements to improve the imaging quality of OEIT. Aiming at the problem of poor image reconstruction quality and low reconstruction accuracy of OEIT, Wang et al. [18] proposed a novel image reconstruction method based on conformal transformation. By this method the imaging quality and accuracy of OEIT are improved, especially for the inclusions located far from the electrode area.

For two-dimensional OEIT, the most used sensor is the uniform sensor that uses electrodes of the same size and places the electrodes evenly at the boundary of the open field [17]. This simple electrode configuration allows the observation of conductivity changes in the open field. However, previous studies showed that the reconstruction quality of uniform sensors for inclusions near the electrode region and those far from the electrode was relatively poor [18]. When the sensor array and the excitation strategy are fixed, the electric field distribution in the observation field is uniquely determined. Therefore, the most fundamental solution to improve the poor quality of OEIT imaging is to optimize the electrode configuration, as well as the excitation strategy.

In this research, a novel focusing sensor is designed based on shape conformal theory to improve the imaging quality of OEIT in the near-electrode region. The conformal bijection is used to map a standard EIT sensor defined at a unit circle to a focusing OEIT sensor defined at an upper half plane. To evaluate the proposed focusing sensor, a series of simulation and experiment analyses are carried out. The results from the novel OEIT sensor are compared with those of a conventional OEIT sensor.

## 2. Materials and Methods

### 2.1. Principle of OEIT

**Figure 1:**
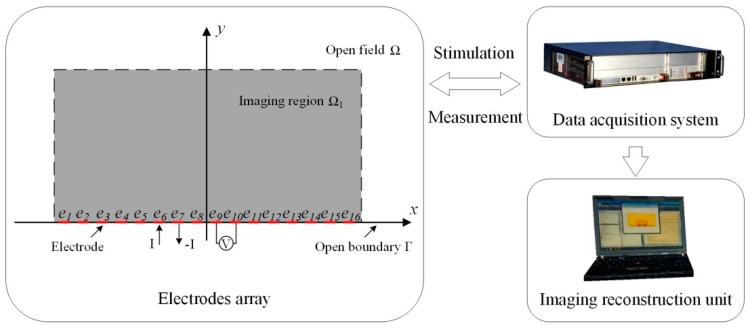
Illustration of the OEIT system with conventional sensor structure.

Figure 1 gives an illustration of an OEIT system with the conventional sensor structure discussed in this paper. The EIT system includes an electrode array, data acquisition system and image reconstruction unit (usually in a PC). The electrode array is uniformly fixed at the boundary Γ of the open domain Ω. The excitation strategy of OEIT system is the conventional adjacent current stimulation and adjacent voltages measurement [19]. At each measuring timepoint, one of the adjacent electrode pairs is excited with currents, and voltage measurements are made between the other adjacent pairs not involving the driven electrodes. The process will continue until all the electrodes have been excited. For a system consisting of *L* electrodes, *L* × (*L*−3) voltage measurements are collected. Based on the reciprocal theory, half of them are independent. Aiming at the sensor with 16 electrodes in this research, there are 208 measurement datapoints in all, and the independent data is the half of this number. When low-frequency currents are applied to the active electrodes, the electric potential *φ*(*x*, *y*) at Ω satisfies the Gaussian equation:

(1) ∇⋅[σ(x,y)∇ϕ(x,y)]=0,  {(x,y)|x∈(−∞,+∞),y∈(0,+∞)},

where *σ*(*x*, *y*) is the conductivity distribution. Following the complete electrode model (CEM) [20], the boundary condition of the OEIT forward problem can be written as follows:

(2) $$\{\begin{array}{c} \sigma (x,y)\frac{\partial \phi (x,y)}{\partial y}{|}_{y=0}=0,\;\;x\in \Gamma \backslash \overset{L}{\underset{l=1}{\cup }}e_{l}\\ \phi (x,y)+\rho_{l}\sigma (x,y)\frac{\partial \phi (x,y)}{\partial y}{|}_{y=0}=U_{l},\;\;x\in e_{l},l=1,2,\ldots ,L\\ \int_{e_{l}}\sigma (x,y)\frac{\partial \phi (x,y)}{\partial y}{|}_{y=0}ds=I_{l},\;\;l=1,2,\ldots ,L\\ \sum_{l=1}^{L}I_{l}=0\\ \sum_{l=1}^{L}U_{l}=0 \end{array}$$

where Γ∈(−∞,+∞) is the actual boundary of open domain, *e_l_* denotes the electrode *l*, *ρ_l_* is the contact impedance between electrode *l* and internal medium, *U_l_* is the voltage on the electrode *l*, *I_l_* is current injected into field Ω from electrode *l*.

The conventional method to calculate the OEIT problem is to simplify the infinite observation domain Ω to a finite domain Ω_1_ [17]. However, this method will lead to truncation errors not only in the forward problem solution but also the inverse problem solution. In our previous research, a transformation domain method was proposed to directly solve the OEIT problem in the infinite observation domain Ω. According to the Riemann mapping theorem, the infinite upper half plane can be transferred into a simple closed circular domain by bijective holomorphic mapping [21]. After conformal mapping, the voltage potential function and the conductivity distribution function still satisfy the Laplace equation and are related to the original equation before transformation. The boundary condition can be calculated by CEM and it has the same form as (2). According to the conformal transformation, the electrical field distribution of the infinite upper half plane can be evaluated by calculating the electrical field distribution in the conformal circular domain. Thus, the process of the imaging method based on conformal transformation has the follow steps:

(1) The boundary voltage measurement data is collected by the electrodes placed on the boundary of the open field.
(2) The reconstructed image is obtained in the conformal circular domain according to the boundary voltage measurement.
(3) The conductivity distribution image of the open field is mapped from the conformal circular domain by the mapping relation [18].

### 2.2. Focusing Sensor Design

**Figure 2:**
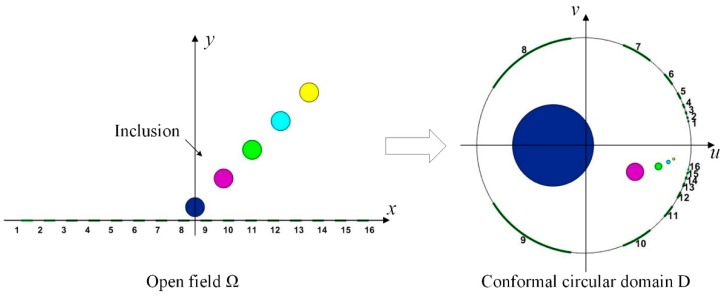
Mapping of inclusions and electrode positions after conformal transformation based on conventional sensor structure.

In previous studies, for two-dimensional OEIT, the most commonly used sensor is the uniform sensor. As shown in Figure 1, electrodes of the same size are fixed at the boundary of the observation domain. This simple electrode configuration allows the observation of conductivity changes in the open field. However, in previous studies, we found that the reconstruction quality of uniform sensor for inclusions near the electrode region and those far from the electrode was relatively poor [18]. Figure 2 shows the mapping of inclusions and electrode positions after conformal transformation based on the conventional uniform sensor structure. Five inclusions of the same size are located at different locations in the observation field. As the inclusions move away from the electrode, their mapping moves to the right semicircular domain, and the relative size increases first and then decreases rapidly. When the uniform sensor in the open field is mapped to the conformal circular domain, the electrode position will be offset, resulting in a sparse electrode distribution on the boundary of the left semicircle domain. When the inclusion near the electrode area is mapped to the conformal circular domain, it is located in the left semicircular domain and the size is relatively enlarged. In this situation, the uniform sensors do not provide a good reconstruction of the inclusions. Therefore, we focus on the high resolution reconstruction in the near electrode region and a focusing sensor optimization scheme based on conformal transformation is proposed.

According to the conformal transformation method, the point (*x*, *y*) of the infinite upper half plane Ω can be mapped to the point (*u*, *v*) in unit circular domain D one by one, the transformation mapping is [22]:

(3) $$w=\frac{\left(1+z\right)}{\left(1-z\right)}i$$

where z=x+yi and w=u+vi. In the meantime, the point (*u*, *v*) in unit circular domain can be mapped to point (*x*, *y*) in the infinite upper half plane by:

(4) $$z=\frac{w-i}{w+i}$$

**Figure 3:**
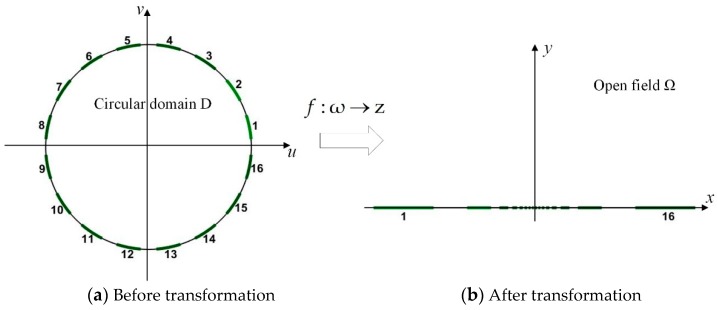
Conformal transformation from unit circular domain to upper half plane.

The realization of the proposed focusing sensor is to map the uniform sensor distribution in the circular domain to the infinite upper half plane through the above mapping relations. Figure 3 shows the process of conformal transformation from the unit circular circle domain to upper half plane. After conformal transformation, the electrode arrangement of focusing sensor expands from the center to both sides, and the electrode spacing also expands. 

Since the sensor with uniform distribution in the circular region also has a higher sensitivity distribution in the left semicircle, the proposed focusing sensor in the open region can improve the sensitivity to the change of conductivity in the near electrode region, which is conducive to the realization of high-resolution reconstruction in the near electrode region.

### 2.3. Forward Problem Solutions

For the solution of EIT, the forward problem is to calculate the boundary voltages from a given conductivity distribution. The forward model is established in EIDORS [23] and solved by the BEM method [24,25]. For the uniform sensor, 16 × 6 mm wide electrodes are evenly placed at the range of [−10, 10] of *x*-axis and the region of imaging is set to be [−10, 10] of *x*-axis and [0, 10] of *y*-axis. For the proposed focusing sensor, according to the above mapping relation, different electrode widths are calculated, and the electrodes are arranged at the center boundary of the open boundary. The region of imaging is set to be the same as the uniform sensor.

### 2.4. Inverse Problem Solutions

**Figure 4:**
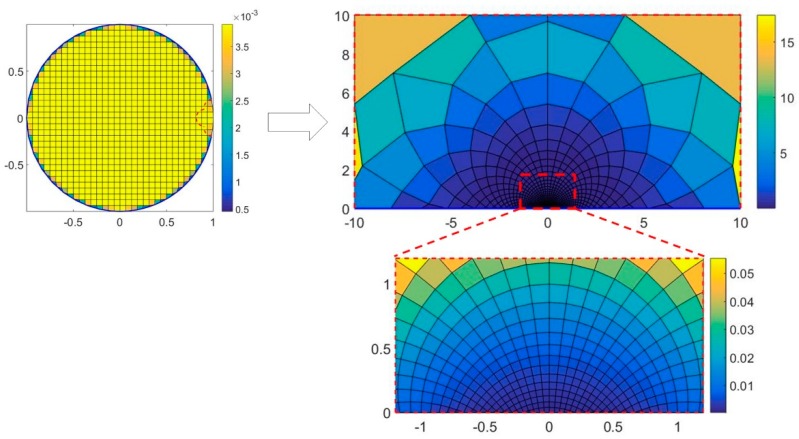
Inverse problem mesh based on conformal transformation.

The inverse problem is solved by FEM method [26]. As shown in Figure 4, the mesh of the open image region is transformed from the uniform mesh in circular field according to the conformal mapping relation. The imaging region is a part of open field, thus the mesh is also a part of the circular field mesh. The color in the figure indicates the area of different grids. The total number of mesh is 850 grids. In this way, the mesh density in the near-electrode region can be greatly improved, which is helpful to realize high-precision image reconstruction.

The inverse problem is to estimate the conductivity distribution from boundary voltage measurements. In EIT, absolute imaging and difference imaging are commonly used for image reconstruction. In this research, the difference image approach is used, which helps to suppress the effect of contact impedance. The reconstruction problem is not well-posed as there is not a single valid solution and small changes in input mean big changes in the output. Also the physics of electric field means that the inverse problem is also ill-conditioned [27]. In this condition, regularization methods have been proved to work well towards these problems which usually have a formal minimization objective function as:

(5) $${\left\|Jh-b\right\|}_{2}^{2}+\lambda G(h)$$

where *J* is the sensitivity matrix, *b* is the difference of the boundary voltage measurement vector between the reference field and the object field, *h* is the conductivity distribution to be solved, *λ* is the regularization factor. Tikhonov regularization algorithm [28] is a widely used non-iterative regularization algorithm. The penalty term of Tikhonov regularization is $G(h)={\left\|h\right\|}_{2}^{2}$. According to the Gaussian Newton method [29], the conductivity image *h* is estimated by the following one-step reconstruction:

(6) $$h={\left(J^{T}J+\lambda I\right)}^{-1}J^{T}b$$

where *I* is an identity matrix. The image reconstruction quality depends on the selection of the regularization factor *λ*. The value is used to affect how much the penalty term controls the final solution. Although there exists the theoretical optimal solution of *λ*, they are usually computational intensive and cannot be used for the real-time imaging. Here, the selection of the regularization factor *λ* is mainly based on the empirical method.

## 3. Results

The proposed focusing sensor is evaluated by both numerical simulation and experimental study. Simulation is carried out using Matlab with EIDORS on a PC equipped with a 3.2 GHz Intel Core i5 processor.

### 3.1. Numerical Simulation

#### 3.1.1. Boundary Measurement Consistency in Transformation Domain

**Figure 5:**
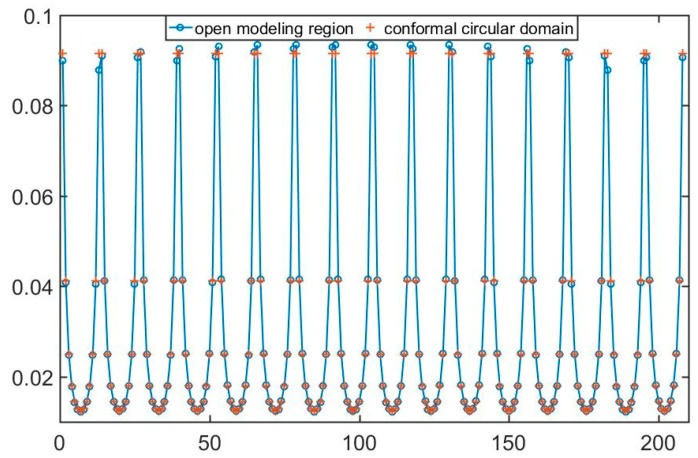
Boundary potential of reference field based on the open modeling region and its conformal circular domain.

In order to simulate the open region, the forward model should be very large to satisfy the potential distribution of an open area. Due to the limitation of the computational cost and the actual application, the forward model can’t be infinite. Considered the simulation precision and calculation cost, according to the previous research in [18], for the forward problem, the modeling region is set to be [−40, 40] on the *x*-axis and [0, 40] on the *y*-axis. Figure 5 shows the boundary potential measurements of the reference field based on the open modeling region and its conformal circular domain. The blue line with circle dot is the boundary potential of reference field based on the open modeling field, while the red dots are the boundary potential based on the conformal circular domain. It can be seen that the boundary measurements of open field are almost consistent with the conformal circular domain. It can be proved that the focusing sensor has same physical properties as its conformal circular domain. At the same time, because the focusing sensor is mapped by the uniform sensor in the conformal circular domain, its boundary potential measurements of each channel is consistent.

**Figure 6:**
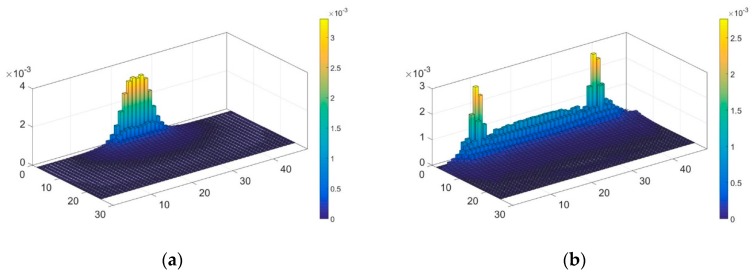
The distribution of sensitivity fields based on (**a**) focusing sensor and (**b**) uniform sensor.

The distribution of sensitivity fields based on the focusing sensor and the traditional uniform sensor are illustrated in Figure 6. The sensitivity distribution indicates the sensitivity of the boundary voltage response to the variation of conductivity at every pixel in the imaging region. The sensitivity at a certain imaging pixel is defined as the Euclidean norm of the Jacobian values at this pixel [30]. The large values represent the change of conductivity at this pixel will cause a large change in boundary voltage response, while the small values represent a low sensitivity to the conductivity variation. As shown in Figure 6, the sensitivity distribution is mapped from the conformal circular domain. It can be seen that the sensitivity distribution of the focusing sensor has higher sensitivity in the central region near the electrodes, and the sensitivity is decreasing rapidly with respect to the direction away from the electrodes. As for the sensitivity distribution of the uniform sensor, the area near the boundary on both sides of the electrodes has high sensitivity. However, the sensitivity distribution in the region near the central electrode is relatively low. According to the comparative result, the focusing sensor is much more sensitive in the central region compared with the uniform sensor. That means the focusing sensors make it easier to detect small changes in electrical conductivity near the central electrode region. This has certain significance for improving the resolution of OEIT detection in the central region near the electrodes.

#### 3.1.2. Quantitative Index

**Figure 7:**
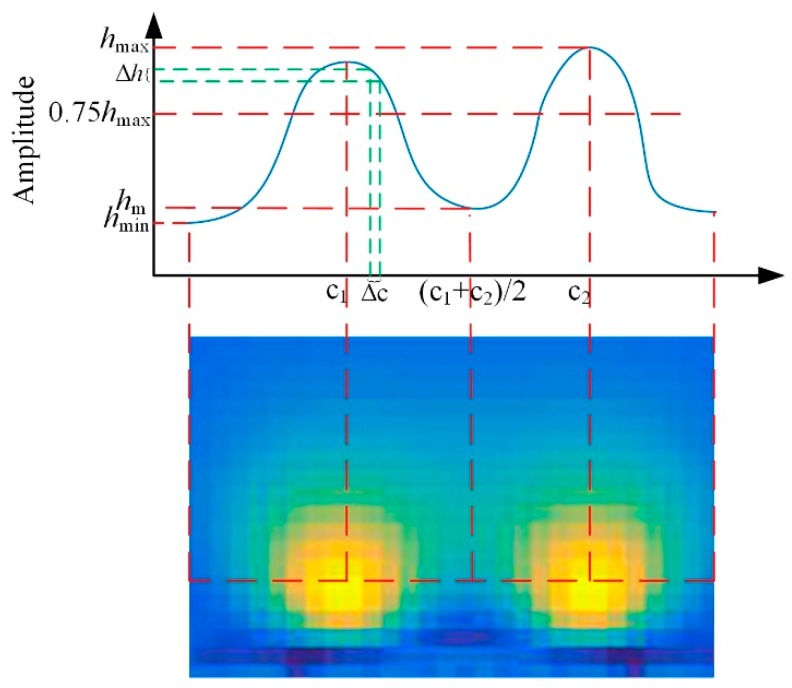
The schematic diagram of Ra and Ag with two inclusions.

To characterize the performance of the focusing sensor compared with the uniform sensor, a series of simulation test are carried out. Then a series of metrics were used to assess the quality of the reconstructed images. The descriptions of these metrics are as follows:

- Resolution (RES) measures the ratio of the number of pixels in inclusions to the total number of pixels, as shown in (7). In the reconstructed images, the reconstructed inclusions are segmented from the reconstructed images by using a threshold of 75% maximum pixel amplitudes [31]. The total number of pixels represents the area of the region of interest for imaging. In this article, RES is used to measure the relative resolution of the focusing sensor and conventional uniform sensor. A smaller RES means the reconstructed image has high resolution for a similar inclusion:
  (7) $$\mathrm{RES}=\sqrt{\frac{Aq}{An}},$$
  where *Aq* is the number of pixels in inclusions, *An* is the total number of pixels of the image region.
- Resolution ability (Ra) is defined as the measurement ability of distinguish between multiple inclusions. Figure 7 illustrates the schematic diagram of Ra with two inclusions as an example. The bottom row shows the reconstructed image, while the top row plots the amplitude across a row through the center of the multiple simulation inclusions. Ra compares the relationship between the amplitude at the midpoint of the centers of the two inclusions and the 75% maximum pixel amplitudes to measure whether the two inclusions can be clearly distinguished in the reconstructed image. The definition of Ra is shown in (8):
  (8) $$\mathrm{Ra}=\frac{h_{m}-0.75h_{\max}}{h_{\min}-0.75h_{\max}},$$
  where *h_m_* is the amplitude value at the midpoint of the centers of the two inclusions, *h*_max_ and *h*_min_ is the maximum and minimum value of the amplitude across the row through the center of the multiple simulation inclusions. For a certain reconstructed image, a Ra greater than 0 indicates that multiple inclusions can be distinguished; a Ra closer to 1 indicates a stronger ability to distinguish inclusions; while a Ra less than 0 indicates that multiple inclusions cannot be distinguished.
- Average gradient (Ag) is defined to measure the steepness of the multiple reconstructed inclusions boundary. As shown in Figure 7, Ag calculates the mean of the absolute value of gradient of the reconstructed pixels between the centers of the multiple simulation inclusions. The definition of Ag is shown in (9):
  (9) $$\mathrm{Ag}=\mathrm{ave}\left(\sum_{s=1}^{t}\left|\frac{h(c_{1}+s\Delta c)-h(c_{1}+(s-1)\Delta c)}{\Delta c}\right|\right),$$
  where $t=\frac{c_{2}-c_{1}}{\Delta c}$. Δc is the length between adjacent pixels. A large Ag means a large relative steepness of the reconstructed inclusion boundary.

In the meanwhile, the relative image error (RE) and the correlation coefficient (CC) between the true conductivity distributions and reconstructed images are also determined. The definitions of the RE and CC can be seen in (10) and (11), respectively [32]:

(10) $$\mathrm{RE}=\frac{\left\|h_{q}-h\right\|}{\left\|h\right\|}\times 100\%,$$

(11) $$\mathrm{CC}=\frac{\sum_{i=1}^{P}\left(h_{i}-\bar{h})(h_{qi}-{\bar{h}}_{q}\right)}{\sqrt{\sum_{i=1}^{P}{\left(h_{i}-\bar{h}\right)}^{2}\sum_{i=1}^{P}{\left(h_{qi}-{\bar{h}}_{q}\right)}^{2}}},$$

where *h_q_* is the normalized conductivity vector of reconstructed distribution, *h_qi_* is the *i*-th element in the vector *h_q_*; and *h* is the normalized conductivity vector of a true distribution, *h_i_* is the *i*-th element in the vector *h*; P is the length of the conductivity vector. For a good reconstruction performance, RE should be low and CC should be relatively high.

#### 3.1.3. Reconstruction Analyses of Multiple Positions

**Figure 8:**
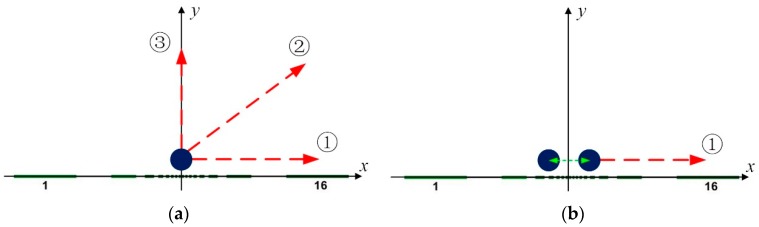
Traces of inclusions involved in dynamic tests: (**a**) single inclusion; (**b**) double inclusions.

During the simulation tests of a single target, highly conductive circular inclusions with diameter of 2–10% of the region width are simulated. The evolutions of the target inclusion with respect to the vertical, horizontal and oblique positions are shown in Figure 8a. In each direction, the inclusions of each size traversed 50 different positions. Direction 1 is the evolution of the horizontal direction near the electrode region and the central coordinate change range is from (0, 1) to (8.5, 1). Direction 2 shows the evolution along the diagonal direction of the field domain and the end of the central coordinate is (−8.5, 1.5). Direction 3 is the evolution keeping away from the center electrode along the vertical direction, and the central coordinates of the inclusions change from (0, 1) to (0, 8.5). During the simulation of double targets, two high conductivity circular inclusions with diameter of 6% of the region width are simulated. The evolution of the target inclusions is along the horizontal direction, and 40 positions are simulated along that direction, as shown in Figure 8b. At each location, the distances between the centers of two inclusions were selected as 0.7, 1.1, and 1.5, respectively, and the degrees of differentiation of the two inclusions measured by different sensors were calculated at each location along with the distance of the inclusions.

**Figure 9:**
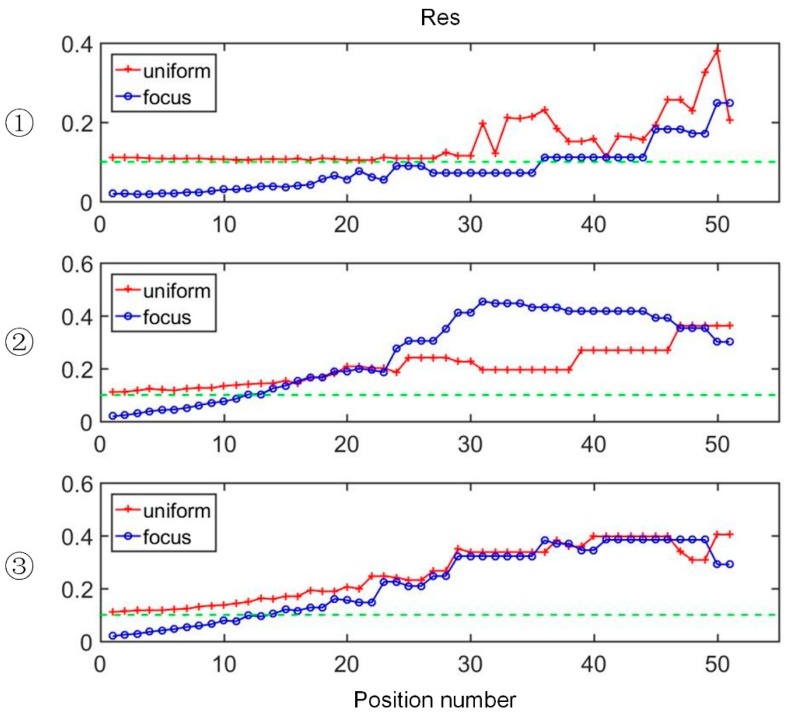
The evolutions of minimum RES with respect to the inclusion positions: ①, ② and ③ denotes the evaluations at Direction 1, 2 and 3, respectively.

Figure 9 shows the evolutions of minimum RES with respect to the inclusion positions in the single target simulations. The minimum RES means the resolution of the smallest inclusions that can be reconstructed by two sensors for different sized inclusions for the same location. A small RES indicates that the sensor is better at recognizing small sized inclusions. The red lines show the minimum RES performance of the uniform sensor, while the blue lines are the performance of the proposed focusing sensor. The green dotted line indicates the 10% resolution, which is usually considered as the normal resolution for EIT. The solving process uses the Tikhonov regularization algorithm. The regularization parameters are determined by an empirical method and they are invariant during each evolution process. As shown in Figure 9, in Direction 1, along with the position of the target moving to the side boundary of the region, the performance of the focusing sensor has more stable and smaller RES, it is better than the uniform sensor in almost all cases. When the inclusions are located in the central region, the resolution of the uniform sensor for the small inclusions is almost stable at 10%, while the minimum resolution of the focusing sensor is up to 2%. During the traversal process of Direction 1, the minimum RES of the focusing sensor is significantly smaller than that of the uniform sensor, and is less than 10% at most positions. This indicates that the focusing sensor can realize high resolution reconstruction of small size inclusions in the near-electrode region, that is, smaller size inclusions can be reconstructed by the focusing sensor. As the inclusion moves from the center to the boundary and gradually away from the electrode with respect to the oblique direction, as shown in Direction 2, a similar conclusion can be reached. When the inclusions are close to the electrode area, the minimum resolution of the focusing sensor has a significant advantage, which is basically less than 10%. However, as the inclusion moves away from the electrode and is located near the side of the imaging area, the focusing sensor gets worse results than the uniform sensor. That means the focusing sensor has poor resolution for inclusions far from the electrode. During the evolution of Direction 3, it can be seen that the focusing sensor has smaller RES than the uniform sensor when the inclusion is near the electrode, which means the focusing sensor can reconstruct smaller inclusions in this situation. When the inclusion moves away from the central electrode, the difference in RES between the two sensors is decreasing. To sum up, the proposed focusing sensor has significant advantages in the reconstruction of small inclusions near the electrode area compared with the uniform sensor. In the simulation tests, the minimum RES of the focusing sensor can up to 2%, which is a significant improvement over conventional uniform sensors. Meanwhile, as shown in Figure 9, when the position number is lower than 15, it can be seen the minimum resolution of the focusing sensor in different traversal directions is better than the uniform sensor, which roughly determines that the high-resolution imaging area of the focusing sensor is [−2.5, 2.5] on the *x*-axis and [0, 2.5] on the *y*-axis. However, the results also show that the focusing sensor has poor resolution for the inclusions that are far away from the central electrode region and near the boundary.

**Figure 10:**
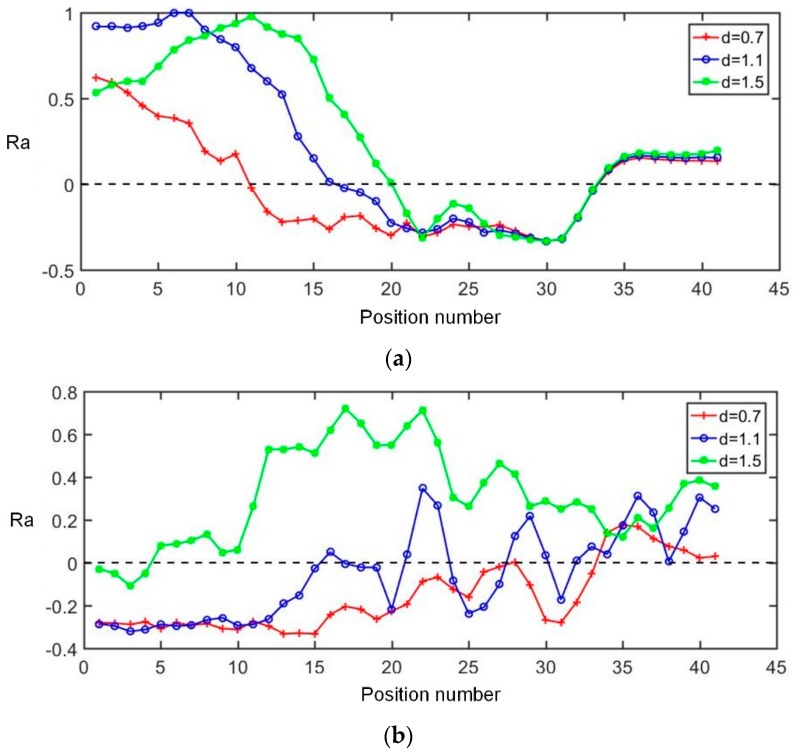
The evolutions of **Ra** with respect to the inclusion positions based on (**a**) focusing sensor and (**b**) uniform sensor.

Figure 10 shows the evolution of Ra with respect to the inclusion positions in the double targets simulations. This simulation compares the ability of two sensors to distinguish multiple inclusions in the near-electrode region. In Figure 10, the top image is the Ra of the focusing sensor, while the bottom image is the Ra of the uniform sensor. The *d* is the distance between the centers of two targets. The horizontal black dotted line indicates the zero value of Ra. If the value is above 0, the higher the value is, the stronger the ability to distinguish between the two inclusions is. If the value is lower than 0, it cannot distinguish the two inclusions. As it can be seen from Figure 10, when the inclusions are located near the central electrodes region, the focusing sensor can distinguish the two inclusions with a center distance of 0.7, while the uniform sensor can only distinguish the two inclusions with a center distance of 1.5. As the inclusions move away from the central region, the ability of the focusing sensor to distinguish close inclusions gradually weakens. However, the uniform sensor can hardly distinguish two inclusions that are close to each other. When the position number is lower than 15, the Ra of the focusing sensor is better than the uniform sensor, which is consistent with the simulation results of the minimum resolution of a single inclusion. In summary, the proposed focusing sensor, compared with the uniform sensor, has a high resolution in the near center electrode region and a strong ability to distinguish the two inclusions in close proximity. According to the above simulation results, the focusing region of the proposed focusing sensor is about [−2.5, 2.5] on the *x*-axis and [0, 2.5] on the *y*-axis.

**Figure 11:**
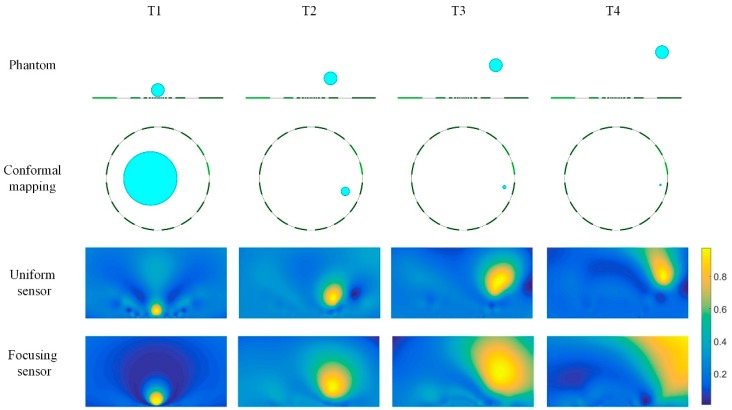
Reconstruction results of single target based on uniform and focusing sensors.

Figure 11 shows the reconstructed results of the single circle target with same size distributed at different positions that are gradually away from the electrode with respect to the oblique direction. In this simulation, the conductivity of the single circle target is 2 S/m, while the background conductivity is 1 S/m. In the meanwhile, Figure 11 also gives the position of the phantom in the conformal circular domain. Compared the reconstructed results of the focusing sensor and the uniform sensor, it can be shown that when the target positions are close to the central electrodes (T1), the focusing sensor leads to better reconstructed results both in location and shape of the circle target and has the highest reconstruction image quality, while the reconstructed image of the uniform sensor has some distortion for the size and shape of the circle target. As the target moves away from the central electrode and close to the boundary, the reconstructed image of focusing sensor becomes bad and gradually worse than that of the uniform sensor. Therefore, the subsequent simulation focused on the comparison of the imaging results of the two sensors in the area near the central electrodes.

**Figure 12:**
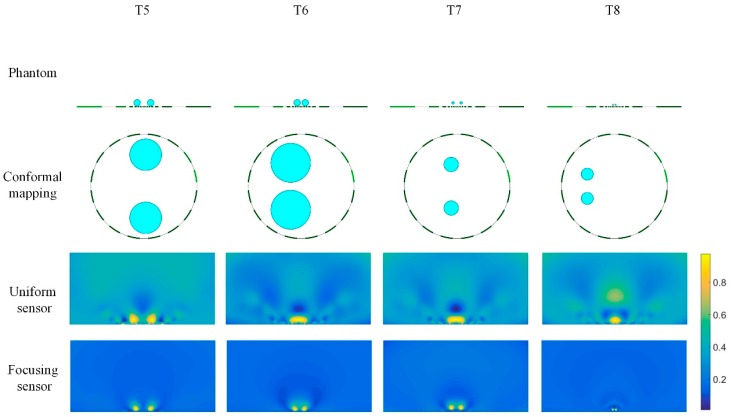
Reconstruction results of double targets based on uniform and focusing sensors.

**Figure 13:**
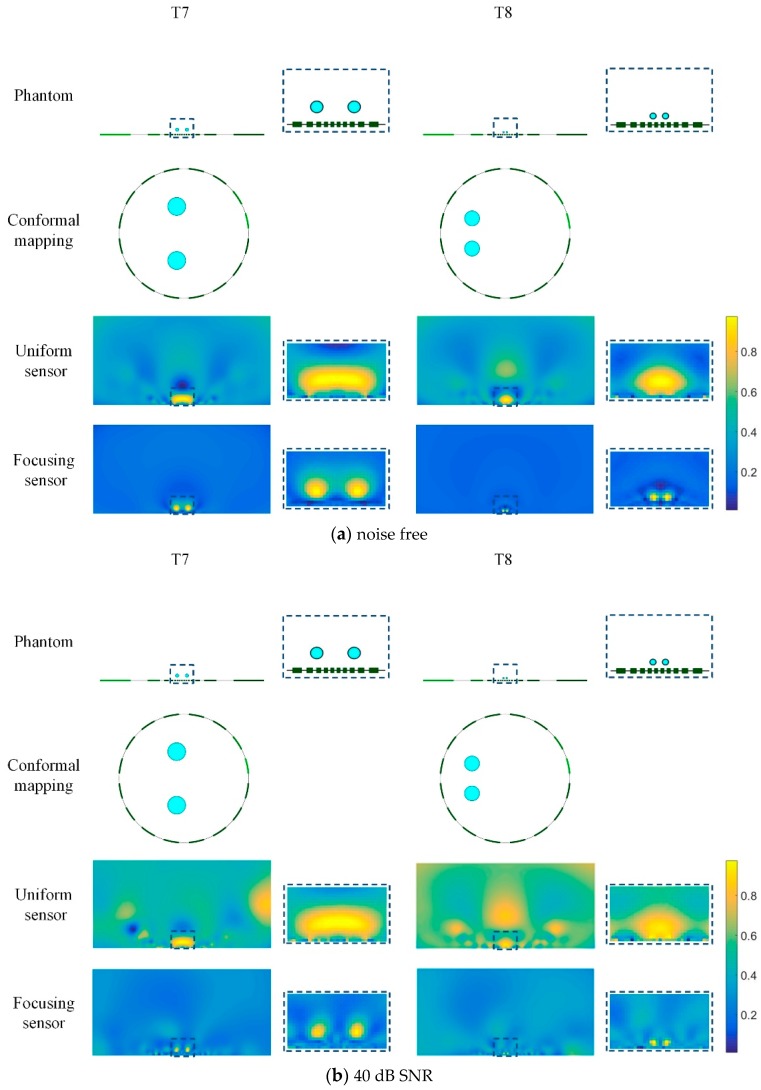
Reconstruction results of double targets based on uniform and focusing sensors with noise free data and 40 dB SNR data.

Figure 12 shows the reconstructed results of double targets with different size and different distance based on the uniform sensor and the focusing sensor. T5 and T6 are two conductive inclusions with same radius of 0.5 and at different distance from each other. It can be seen that when two inclusions are far apart (T5), both sensors can distinguish the two inclusions. When the two inclusions are close to each other, they cannot be distinguished in the reconstructed image of the uniform sensor. In this case, the reconstructed results of uniform sensor can only reflect the presence of the inclusions, but does not distinguish the number and size of the inclusions. However, from the results of the focusing sensor, he number and size of the inclusions can be distinguished clearly. Then the size and distance of the inclusions are gradually decreased, as shown in T7 and T8. Due to the small size of the inclusions, the distribution of T7 and T8 and the reconstruction results are locally amplified in Figure 13a. T7 shows two conductive inclusions with same radius of 0.2 and relatively close to each other. It can be found that the uniform sensor cannot get a good performance for the two small inclusions and the reconstructed image is similar to the image of T6, which means the uniform sensor cannot distinguish the two small inclusions. However, the focusing sensor has a great performance in this case and the number and size of the inclusions can be clearly distinguished. T8 is two conductive inclusions with the same radius of 0.1 and closer to each other. In this case, the uniform sensor can only reconstruct a large region of conductivity changes and the exact distribution of the inclusions is unknown. On the contrary, the focusing sensor still has great reconstruction quality, and it can represent well not only the size of the inclusions, but also the location of the inclusions. In this case, the focusing sensor has a minimum resolution of 2%.

A quantitative analysis of these reconstruction results is shown in Table 1. As it can be seen from the table, in all four cases, the results of the focusing sensor have better Ra than the uniform sensor. Except for T5, the Ra of the uniform sensor is negative, which means the uniform sensor cannot distinguish small inclusions that are close to each other. Ag measures the steepness of the multiple reconstructed inclusions boundary. When the inclusions are far from each other, the Ag values obtained by the two sensors are similar. When the size and distance of the inclusions are gradually decreased, the reconstruction results of the uniform sensor can no longer distinguish the two inclusions, and then Ag of the focusing sensor becomes much larger than that of the uniform sensor. Therefore, the focusing sensor has strong resolution ability for the inclusions located in the area nearby the central electrodes. Meanwhile, the results based on the focusing sensor have lower RE and higher CC in almost all simulated situations. This means that the images of the focusing sensor show better image quality and reconstruction accuracy. Moreover, as the inclusion size and spacing decreases, the advantages of focusing sensor become more and more obvious.

In order to explore the performance of different sensors in the presence of noise, T7 and T8 are simulated in the case of no noise and 40dB SNR noise, respectively. The result is shown in Figure 13. The uniform sensor results have deformations in the shape of the inclusions and cannot distinguish the two inclusions. On the contrary, the result of the proposed focusing sensor has less deformation and can reflect the actual circumstances of the circle target. In the meantime, when 40 dB SNR random noise is added to the simulation data the uniform sensor leads to bad results in which the reconstructed target has large distortion and image artifacts. However, under the influence of noise, the focusing sensor can reconstruct the true distribution of inclusions, especially for small inclusions close to each other.

Table 2 shows the quantitative analyses of these reconstructed results. The results of the proposed focusing sensor have lower RE and higher CC in all of the tested phantoms compared with the uniform sensor results. In these cases, the Ag of the focusing sensor is much better than the uniform sensor and the Ag becomes larger as the size and distance of the inclusions decrease. This indicates that the boundary of the inclusions reconstructed by the focusing sensor is steep, and the steepness increases with the decrease of the distance between inclusions. Meanwhile, the Ra of the proposed focusing sensor are obviously better than the uniform sensor results, which means that under the influence of noise, the focusing sensor can still reconstruct multiple small inclusions located at the area near the central electrodes. Moreover, the reconstructed images of the focusing sensor have less image artifacts and higher reconstruction quality compared with the uniform sensor ones.

#### 3.1.4. Reconstruction Results Combining the Focusing Sensor and Uniform Sensor

The former discussion shows the focusing sensor can reconstruct smaller targets near the central electrode, but its reconstructed results for targets far away from the electrodes are worse than those of the uniform sensor. Therefore, if a measurement of the same object was taken using each individual sensor, in the same environment and in the same location then it could be assumed that all of the measurements were from one device rather than individual sensors. The reconstruction could then use all of the combined measurements in order to reconstruct the conductivity distribution. There are two ways to fuse the data from two different sensors. The first method (Method 1) is to directly superimpose the reconstructed images of the two sensors. The second method (Method 2) is to combine all of the measurements from two sensors and then the augmented sensitivity matrix is used to solve the same solution vector. This means that each calculation now uses 2 × 208 measurements, where 208 is the number of measurements of each sensor. This improves the overall solution as more measurements are used to calculate the same number of answers.

**Figure 14:**
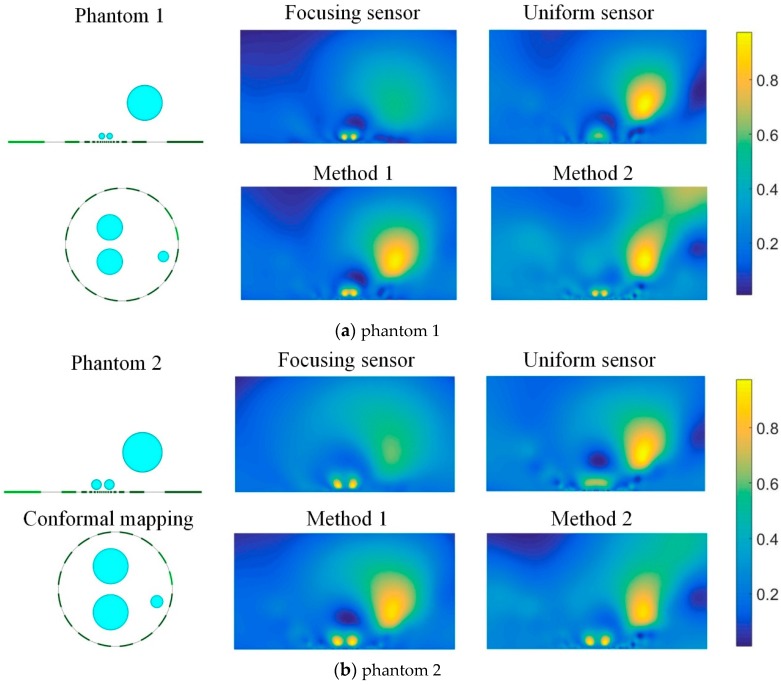
Reconstructed results of two different phantoms from focusing sensor, uniform sensor, and two fusion methods.

Figure 14 shows reconstructed results of two different phantoms based on each individual sensor as well as the two data fusion methods. When there exists a small inclusion close to the electrode and large inclusions far from the electrode, the reconstructed image of focusing sensor can clearly recognize the two small inclusions that are close to the electrode and close to each other, but the large inclusion far from the electrode can hardly be recognized. On the contrary, the uniform sensor can reconstruct the large inclusion far from the electrode, but cannot recognize the two small inclusions that are close to the electrode. Through the data fusion of two different sensors, from the reconstructed image, it can be found that the combination of two measurement data can effectively improve the reconstruction quality of multiple inclusions. Method 1 and Method 2 can both reconstruct the multiple inclusions compared with the reconstructed results of each individual sensor. These results indicate that the fusion of the measurements from two sensors can not only realize high-resolution reconstruction of multiple inclusions near the electrode, but also realize high-quality reconstruction of distant inclusions.

### 3.2. Experimental Analysis

**Figure 15:**
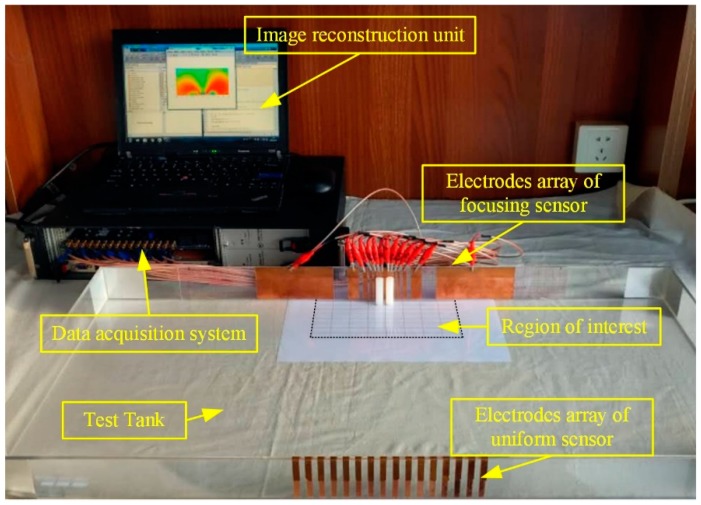
The experimental system for OEIT.

**Figure 16:**
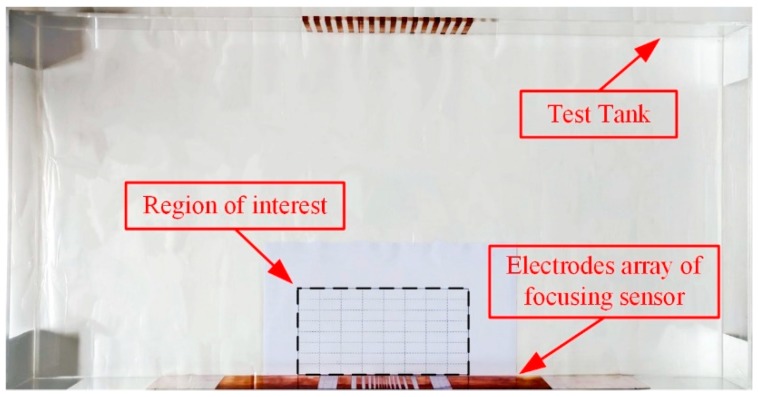
The schematic diagram of the region of interest.

To further test the performance of the proposed focusing sensor, a set of experimental studies are carried out. The experimental equipment is show in Figure 15. It consists of a rectangular tank and a data acquisition system. The EIT system was developed in Tianjin University, China. It is a sixteen-channel high-speed serial data acquisition (DAQ) system [33]. The rectangular tank is 80 cm in length and 40 cm in width, which is used to simulate the open region. For the proposed focusing sensor, the electrodes are 16 copper plates with different widths which are consistent with the simulation settings. They are placed on the central boundary of the rectangular tank. Table 3 shows the actual dimensions of the focusing sensor electrodes used in experimental studies. For comparison, the electrodes of the uniform sensor are 16 copper plates with 6 mm width and they are uniformly placed on the central boundary of the rectangular tank. According to the simulation results, the imaging region is focused on the shallow region near the electrodes. Thus, the region of interest is set to 20 cm in length and 10 cm in width. Figure 16 shows the schematic diagram of the region of interest. The experiments are conducted by placing different size of plastic (nonconductive) or metallic (conductive) rods placed at different locations. Na_2_SO_4_ solution with a conductivity of 6.22 × 10^−2^ S/m is selected as the background medium.

**Figure 17:**
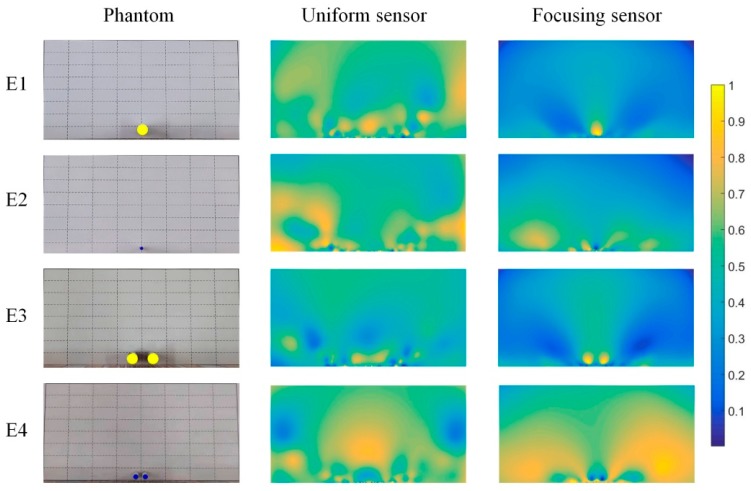
Experimental results from proposed focusing sensor and uniform sensor with Tikhonov regularization.

The experimental results of different phantoms based on the proposed focusing sensor and uniform sensor are shown in Figure 17. The inclusion of E1 is an aluminum rod of 10 mm diameter, while E2 is a nylon rod with 3 mm diameter. The two inclusions are set at the region near the central electrode. The test aims to compare the ability of reconstructing the small inclusion as well as the resolution of the two sensors. From the reconstructed results, it can be found that the focusing sensor shows better performance than the uniform sensor and the recognition ability of conventional uniform sensors for small sized inclusions is poor, even if the inclusions are located near the electrode area. For the 10 mm diameter aluminum rod (E1), the focusing sensor can reconstruct the position and shape of the inclusions with high accuracy, while the uniform sensor can only reconstruct the general area of conductivity variation with large image artifacts. When the size of the inclusions is reduced to 3 mm (E2), the uniform sensor cannot reconstruct the conductivity variation, while the focusing sensor can still reconstruct the inclusion clearly. The above results shows that the proposed focusing sensor is more sensitive to small conductivity changes in the near electrode region and the focusing sensor can improve the reconstruction resolution to at least 3% in the near central electrodes region. E3 and E4 show the experimental results for double inclusions, which is to test the ability of different sensors to distinguish multiple inclusions in close proximity. E3 is two same sizes of 10 mm aluminum rods located near the electrodes. The uniform sensor still can only reconstruct the blurred image and the inclusions are not clearly identifiable. However, the two inclusions can be clearly distinguished from the reconstruction image of focusing sensor, although the reconstructed inclusions have slight deformation. E4 shows two same size nylon rods with 5 mm diameter and closer to the electrodes, the reconstruction result of focusing sensor is significantly better than that of the uniform sensor. The uniform sensor cannot recognize small size inclusions, while focusing sensor can not only recognize two small size inclusions that are lose to each other, but also reconstruct them with a high accuracy. Table 4 shows the quantitative analyses of the experimental reconstruction results. The quantitative analyses shows that the results of focusing sensor can reach higher CC and lower RE compared with the results of uniform sensor for all the experimental reconstruction images. The above results show that the proposed focusing sensor has higher sensitivity and reconstruction accuracy to small size inclusions in the near-electrode region. Compared with the uniform sensor, the focusing sensor has better reconstruction quality and higher reconstruction accuracy for small conductivity changes in the focusing region. The experimental results show that the resolution of the focusing sensor can reach 3% in the near center electrode region.

**Figure 18:**
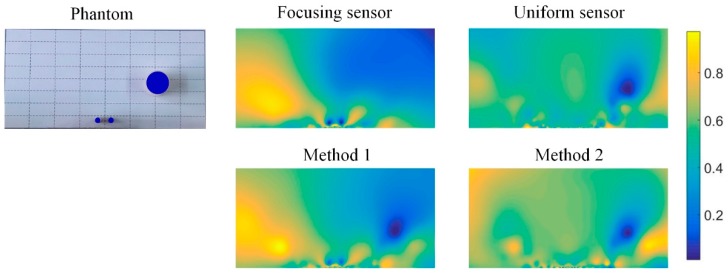
Experimental reconstructions from focusing sensor, uniform sensor, and two fusion methods.

To further test the performance of the proposed focusing sensor and the two different data fusion methods, an experimental phantom is carried out and compared under different methods. The phantom is two 5 mm nylon rods located near the central electrodes and a 25 mm nylon rod far away from the electrodes. Figure 18 shows the reconstructed results based on each individual sensor and the two data fusion methods. The experimental results are consistent with the simulation results. The focusing sensor has high resolution ability in the focusing region. It can reconstruct small targets in the area near the electrodes, but its reconstructed results for the target far away from the electrodes are worse than the uniform sensor. However, the uniform sensor can hardly reconstruct the two small targets when there has a large inclusion far away from the electrodes. The situation is improved through the data fusion method 1. From the results, Method 1 can basically present small inclusions near the electrode and large inclusions far from the electrode. However, the results of Method 2, which has severe deformation and image artifacts, are relatively poor. This is because in the experimental environment, uncertain noises and errors will increase the difficulty of data fusion under different sensors. Therefore, Method 2 adopts the method of solving the same solution matrix, which will increase the ill-posedness of the solution. Method 1 solves the optimal solution under each sensor and then fuses the image. By this method, the conductivity distribution of the measured field can be reconstructed more accurately. Because the data fusion method proposed in this paper combines the advantages of the two sensors, this method may have the possibility of realization in some application fields. For example, in the surface geological exploration and landmine detection, these two sensors could be used to scan the same static object, successively. Then these two kinds of data could be fused to reconstruct the same object.

## 4. Conclusions

A new sensor design based on conformal transformation for OEIT is proposed to improve the image reconstruction quality and low reconstruction accuracy of OEIT. The proposed focusing sensor is derived from the uniform distribution of electrodes in the conformal circular domain through conformal transformation method. The numerical results show that compared with the conventional uniform sensor, the proposed focusing sensor can achieve high resolution reconstruction of the inclusions near the central electrode with a minimum resolution of 2%. The evolution results roughly determine the high-resolution imaging area of the focusing sensor is [−2.5, 2.5] of *x*-axis and [0, 2.5] of *y*-axis. Moreover, the focusing sensor can distinguish small sized inclusions that are close to each other in the focusing region and reconstruct them with high quality. Qualitative and quantitative results show that the results of the proposed focusing sensor have smaller minimum resolution and higher resolution ability when the position of target is near the central electrodes. The experimental results show that the resolution of the focusing sensor can reach 3% in the near center electrode region. The data fusion methods of the two sensors are also discussed. The experimental results show that the method that solves the optimal solution under each sensor and then fuses the image can lead to better results compared with the method of solving the same solution matrix. However, the proposed method can be used for scanning imaging based on a one-dimensional linear electrode array at present, and the method of constructing two-dimensional focusing electrode arrays is still the main topic of future research. Future work will focus on the construction of two-dimensional electrode array as well as the further improvement of the reconstruction algorithms.

## Figures and Tables

**Table 1:** Quantitative analyses of the reconstruction results shown in Figure 12.

	T5		T6		T7		T8	
	Uniform	Focusing	Uniform	Focusing	Uniform	Focusing	Uniform	Focusing
Ra	0.642	0.813	−0.132	0.611	−0.149	0.545	−0.217	0.498
Ag	4.531	6.045	0.728	12.145	0.538	14.266	0.911	28.644
RE	0.132	0.143	0.177	0.101	0.233	0.131	0.250	0.089
CC	0.855	0.872	0.780	0.842	0.750	0.851	0.714	0.887

**Table 2:** Quantitative analyses of reconstruction results shown in Figure 13.

	Noise Free				40 dB SNR			
	T7		T8		T7		T8	
	Uniform	Focusing	Uniform	Focusing	Uniform	Focusing	Uniform	Focusing
Ra	−0.149	0.545	−0.217	0.498	−0.161	0.441	−0.117	0.312
Ag	0.538	14.266	0.911	28.644	0.507	13.889	0.912	28.627
RE	0.233	0.131	0.250	0.089	0.251	0.142	0.370	0.136
CC	0.750	0.851	0.714	0.887	0.678	0.788	0.623	0.811

**Table 3:** The actual dimensions of the focusing sensor electrodes used in experimental studies.

Electrode Number	1	2	3	4	5	6	7	8
	(16)	(15)	(14)	(13)	(12)	(11)	(10)	(9)
Length (mm)	110	15.5	6.0	3.5	2.6	2.0	1.7	1.5

**Table 4:** Quantitative analyses of experimental reconstruction results shown in Figure 17.

	E1		E2		E3		E4	
	Uniform	Focusing	Uniform	Focusing	Uniform	Focusing	Uniform	Focusing
RE	0.314	0.150	0.238	0.093	0.271	0.164	0.325	0.137
CC	0.681	0.879	0.713	0.895	0.737	0.828	0.632	0.841
